# Impacted ureteric stone causing iliofemoral vein thrombosis: The first reported case

**DOI:** 10.1016/j.ijscr.2018.10.065

**Published:** 2018-10-31

**Authors:** Mohammed Abed Al Kadum Hassan, Rawa Hama Ghareeb Ali, Okba Fathy Ahmed, Fahmi H. Kakamad, Hewa Mahmood Toffeq

**Affiliations:** a Sulaymaniyah, Surgical Teaching Hospital, Urology Department, Iraq; b Mosul Cardiac Center, Mosul, Iraq; c Sulaymaniyah, Surgical Teaching Hospital, Cardiothoracic Department, Iraq

**Keywords:** Deep vein thrombosis, Ureteric Stone, Ureteroscopy, Case report

## Abstract

**Introduction:**

Extrinsic compression with resulting obstruction of the iliac veins is recognized as a crucial cause of deep vein thrombosis (DVT). We report a case of impacted ureteric stone causing DVT.

**Case report:**

A 65-year-old man presented with left lower limb swelling that diagnosed and treated as extensive DVT. Further evaluation revealed incidental left severe hydroureteronephrosis and impacted ureteric stone compressing iliac vessels. Immediate percutaneous nephrostomy done followed by left ureterolithotripsy 6 weeks later.

**Discussion:**

several iliofemoral venous thromboses have been reported due to extrinsic compression by various pathologies, but to best of our knowledge, this is the 1st report of the impacted ureteric stone causing life-threatening proximal DVT.

**Conclusion:**

Isolated unilateral iliofemoral DVT may raise the suspicion of pelvic pathology including ureteric stone.

## Highlights

• Deep vein thrombosis (DVT) is a relatively common medical problem.
• Extrinsic compression with resulting obstruction of the iliac veins is recognized as a crucial cause of DVT.
• Different pelvic pathologies may cause compression of iliofemoral vein and cause venous thrombosis.
• Impacted ureteric stone has caused the left iliofemoral thrombosis in the current case.

## 1 Introduction

Deep vein thrombosis (DVT) is a relatively common medical emergency occurring in 56–122 per 100,000 individuals annually [1]. Its pathogenesis is usually related to venous stasis (obstructive or nonobstructive), disruption of the vascular wall or hypercoagulopathy [2].

Extrinsic compression with resulting obstruction of the iliac veins is recognized as a crucial cause of DVT [3]. Thrombosis of the left iliofemoral venous system has previously been attributed to the extrinsic compression in the lower abdominal and pelvic region. Causes of compression include but not limited to the pressure effect of right iliac artery (May-Thurner syndrome), a distended urinary bladder, endometriosis, and common and internal iliac artery aneurysms [4]. The aim of this report is to present and discuss a case of impacted ureteric stone that causes deep venous thrombosis. This case has been reported in line with the SCARE criteria [5].

## 2 Case report

**Fig. 1:**
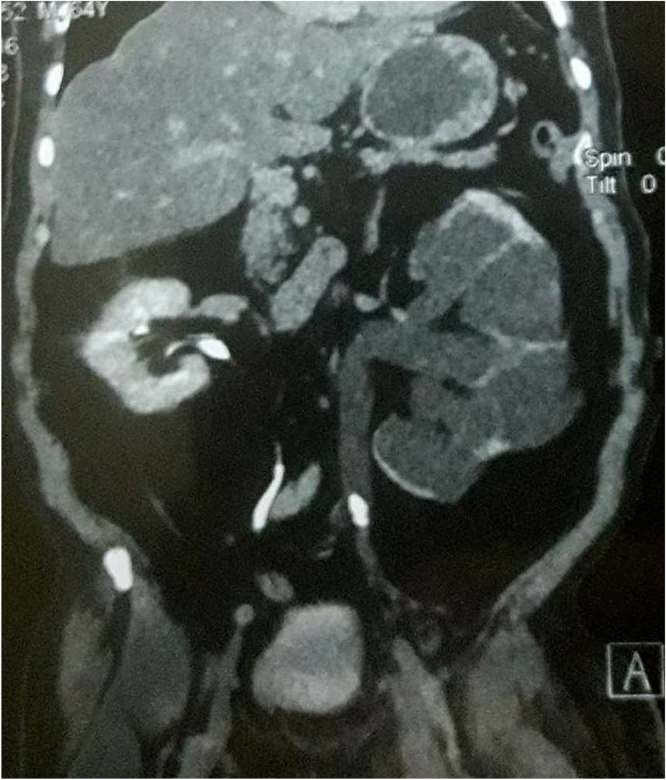
impacted left ureteric stone causing severe left hydroureteronephrosis.

**Fig. 2:**
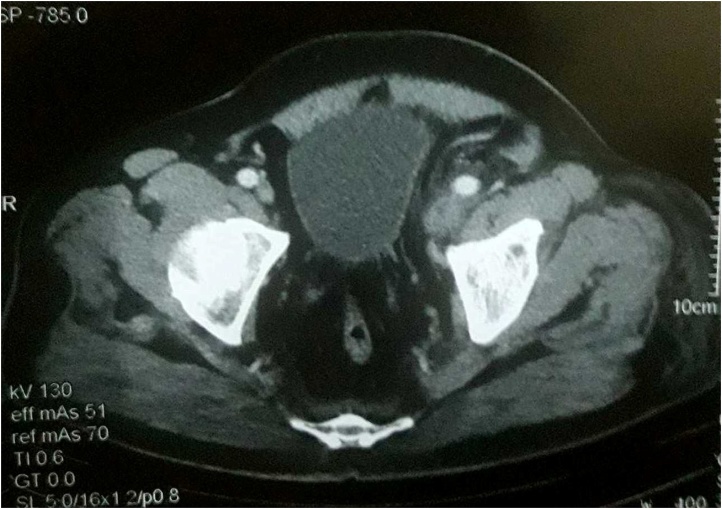
Axial CT scan showing left femoral vein thrombosis.

**Fig. 3:**
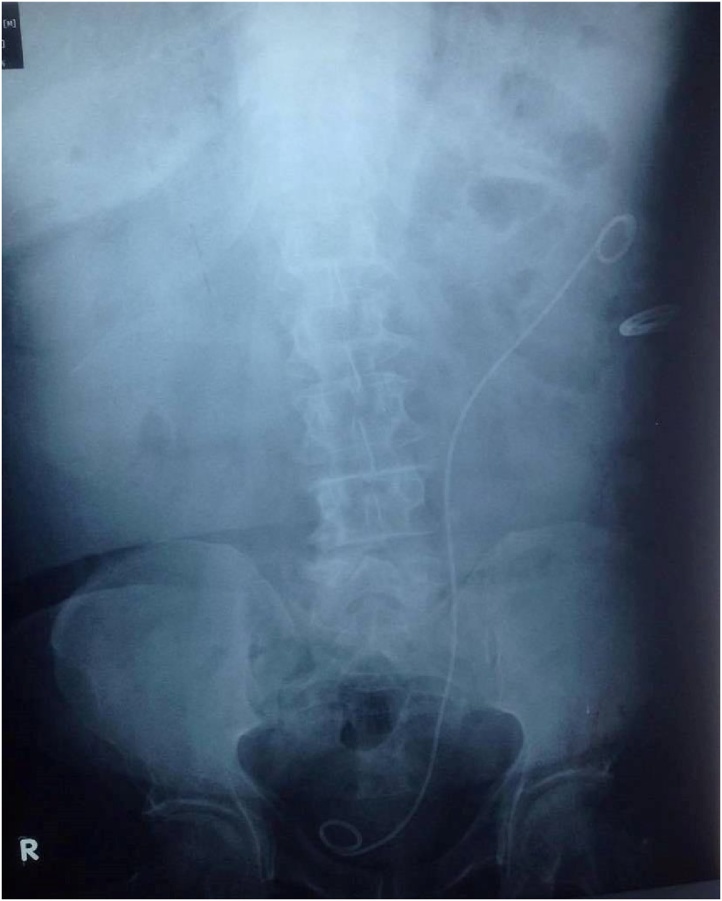
Post operative KUB showing JJ stent and nephrostomy tube.

A 65-year-old man referred to the emergency department for evaluation of the lower extremity swelling associated with pain for four days. Physical examination demonstrated an overweight man (BMI 29.6 kg/m^2^) with extensive pitting edema of the left lower limb from the groin to the knee joint with calf tenderness. Color Doppler ultrasound revealed an extensive DVT involving common iliac, external iliac and common femoral vein as well as superficial femoral down to popliteal vein. He was treated by bed rest, elevation with bandaging of left leg, 6000 IU of low molecular weight heparin subcutaneously twice a day and further evaluation was performed to find the underlying etiology. Investigations including hematological, immunological, biochemical, lipid profile, protein S and protein C were normal. Abdominal and pelvic ultrasound (US) showed incidental finding of severe left hydroureteronephrosis with almost lost of cortical thickness, for that abdominal and pelvic computed tomography (CT) scan revealed marked left-sided hydroureteronephrosis and an impacting stone measuring (18 × 10 × 10 mm) at the level of L5/S1 (Fig. 1) with signs of DVT affecting left iliac and femoral vein below the above mentioned region (Fig. 2). Next day percutaneous nephrostomy was performed to decompress the hydronephrotic kidney. He was kept as an inpatient for one week under observation then after discharged home on oral anticoagulation in the form of rivaroxaban 20 mg daily. Six weeks later, color Doppler US showed complete recanalization of the superficial femoral, popliteal as well as the proximal segment of deep veins of the leg but common iliac, external iliac and common femoral veins and proximal superficial femoral vein were still partially thrombosed. Under spinal anesthesia, left ureterorenoscopy showed an impacted stone at the level of iliac vessel pulsation causing edema and external compression of the iliac vessels. Through pneumatic lithotripsy, the stone was fragmented and JJ stent inserted (Fig. 3). Next day the patient discharged home and continued on taking his antithrombotic treatment (rivaroxaban 20 mg). The JJ stent was removed 3 weeks later. Three months after that, Doppler US showed complete recanalization of iliac vessels.

## 3 Discussion

In a review of normal anatomy, the ureter enters the pelvis, where it crosses anteriorly to the iliac vessels, which usually occurs at the bifurcation of the common iliac artery into the internal and external iliac arteries. Here, the ureters are within five cm of one another before they diverge laterally [6].

Studies have demonstrated that there is an anatomical predilection for the left iliofemoral vein to form thrombi as an acute setting [7]. It was Virchow who ﬁrst recognized that iliofemoral venous thrombosis was much more common in the left leg than in the right leg [8]. It has been postulated that the right iliac artery produces a hemodynamically signiﬁcant left iliac vein compression, although this may represent a normal anatomic pattern [9]. Thiryayi and his colleagues reported a 39-year-old woman presented with signs and symptoms of left lower limb DVT, hematological tests were normal. Abdominal ultrasound revealed seven cm pelvic mass and CT scan demonstrated semi-cystic non-enhancing mass just lateral to the aortic bifurcation compressing left common and internal iliac veins. On exploration and resultant biopsy and immunohistochemistry, it was confirmed that the patient complained from extra-spinal ependymoma [3]. Stevenson et al published their experience with a 46-year male presented with left lower limb pain and swelling for 2-day duration. Initially, he was diagnosed as a case of idiopathic left lower limb DVT as the sonographer failed to recognize abdominal mass. The patient was kept on anticoagulant but the condition remained the same, after three months, another abdominal ultrasound showed left adrenal related mass, subsequent CT scan and magnetic resonant imaging (MRI) confirmed left pheochromocytoma encasing lower aorta common iliac vessels, more extensive on the left side. The patient was treated with complete mass and lower aortic and iliac vessels with aorto-biliac bypass. In the current case, the only possible and logical explanation for the extensive proximal left lower limb DVT is external compression by overlying ureteric stone.

Extrinsic compression leading to iliofemoral venous thrombosis has also been attributed in several cases caused by various pathologies, but to best of our knowledge, this is the 1st report of the impacted ureteric stone causing life-threatening proximal DVT [[10], [11], [12], [13]]. In the present case, the leading cause is compression but also there may be other predisposing factors such as age, obesity, and left side lateralization [6].

## 4 Conclusion

Isolated unilateral iliofemoral DVT may raise the suspicion of pelvic pathology including ureteric stone. In this situation, definitive management should deal with the pelvic diseases.

## Conflicts of interest

There is no conflict of interest.

## Sources of funding

None to be stated.

## Ethical approval

Approval has been given by Ethical committee of Sulaymaiyah Surgical Teaching Hospital.

NO 25.2018.

## Consent

Written informed consent was obtained from the patient for publication of this case report and accompanying images. A copy of the written consent is available for review by the Editor-in-Chief of this journal on request.

## Author contribution

Design and idea: Mohammed Abed Al Kadum Hassan, Rawa Hama Ghareeb Ali, Fahmi H. Kakamad, Okba F. Ahmed and Hewa Mahmood Toffeq.

Drafting: Rawa Hama Ghareeb Ali, Hewa Mahmood Toffeq and Okba F. Ahmed.

Data aquision: Rawa Hama Ghareeb Ali, Okba F. Ahmed, Fahmi H. Kakamad.

Final revision: Mohammed Abed Al Kadum Hassan, Rawa Hama Ghareeb Ali, Fahmi H. Kakamad, Okba F. Ahmed and Hewa Mahmood Toffeq.

## Registration of research studies

Not applicable.

## Guarantor

The corresponding author is the guarantor of submission.

## Provenance and peer review

Not commissioned, externally peer reviewed.
